# 1,3-Bis[4-(dimethyl­amino)benz­yl]-4,5,6,7-tetra­hydro-1*H*-1,3-diazepan-2-ium chloride

**DOI:** 10.1107/S1600536808041603

**Published:** 2008-12-13

**Authors:** Hakan Arslan, Don VanDerveer, Yetkin Gök, İsmail Özdemir, Bekir Çetinkaya

**Affiliations:** a Department of Natural Sciences, Fayetteville State University, Fayetteville, NC 28301, USA; b Department of Chemistry, Faculty of Pharmacy, Mersin University, Mersin, TR 33169, Turkey; c Department of Chemistry, Clemson University, Clemson, SC 29634, USA; d Department of Chemistry, Faculty of Science and Arts, İnönü University, Malatya, TR 44280, Turkey; e Department of Chemistry, Faculty of Science, Ege University, Bornova-İzmir, TR 35100, Turkey

## Abstract

The title *N*-heterocyclic carbene derivative, C_23_H_33_N_4_^+^·Cl^−^, has been synthesized and characterized by elemental analysis, ^1^H and ^13^C NMR, IR spectroscopy and a single-crystal X-ray diffraction study. Ions of the title compound are linked by three C—H⋯Cl inter­actions. The seven-membered 1,3-diazepane ring has a form inter­mediate between twist-chair and twist-boat.

## Related literature

For the synthesis, see: Özdemir *et al.* (2005 ▶); Yaşar *et al.* (2008 ▶). For general background, see: Hermann (2002 ▶); Littke & Fu (2002 ▶); Evans & Boeyens (1989 ▶). For puckering parameters, see: Cremer & Pople (1975 ▶). For related compounds, see: Arslan *et al.* (2007*a* ▶,*b* ▶,*c* ▶). For general background to the use of *N*-heterocyclic carbenes as phosphine mimics and in catalysis, see: Arduengo & Krafczyk (1998 ▶); Dullius *et al.* (1998 ▶); Glorius (2007 ▶); Hermann & Köcher (1997 ▶); Nolan (2006 ▶); Regitz (1996 ▶). For bond-length data, see: Allen *et al.* (1987 ▶).
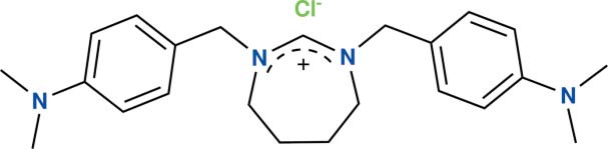


## Experimental

### 

#### Crystal data

C_23_H_33_N_4_^+^·Cl^−^
*M* *_r_* = 400.98
Orthorhombic, 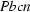

*a* = 22.663 (5) Å
*b* = 10.081 (2) Å
*c* = 9.6368 (19) Å
*V* = 2201.7 (8) Å^3^
*Z* = 4
Mo *K*α radiation
μ = 0.19 mm^−1^
*T* = 153 (2) K
0.46 × 0.12 × 0.07 mm

#### Data collection

Rigaku Mercury CCD diffractometer
Absorption correction: multi-scan (*REQAB*; Jacobson, 1998 ▶) *T*_min_ = 0.918, *T*_max_ = 0.987
14704 measured reflections
1947 independent reflections
1481 reflections with *I* > 2σ(*I*)
*R*_int_ = 0.053

#### Refinement

*R*[*F* ^2^ > 2σ(*F* ^2^)] = 0.059
*wR*(*F* ^2^) = 0.160
*S* = 1.10
1947 reflections
130 parameters
H-atom parameters constrained
Δρ_max_ = 0.28 e Å^−3^
Δρ_min_ = −0.30 e Å^−3^

### 

Data collection: *CrystalClear* (Rigaku/MSC, 2001 ▶); cell refinement: *CrystalClear*; data reduction: *CrystalClear*; program(s) used to solve structure: *SHELXTL* (Sheldrick, 2008 ▶); program(s) used to refine structure: *SHELXTL*; molecular graphics: *SHELXTL* and *Mercury* (Macrae *et al.*, 2006 ▶); software used to prepare material for publication: *SHELXTL*.

## Supplementary Material

Crystal structure: contains datablocks global, I. DOI: 10.1107/S1600536808041603/hg2445sup1.cif

Structure factors: contains datablocks I. DOI: 10.1107/S1600536808041603/hg2445Isup2.hkl

Additional supplementary materials:  crystallographic information; 3D view; checkCIF report

## Figures and Tables

**Table 1:** Hydrogen-bond geometry (Å, °)

*D*—H⋯*A*	*D*—H	H⋯*A*	*D*⋯*A*	*D*—H⋯*A*
C1—H1⋯Cl1^i^	0.96	2.53	3.486 (4)	180
C2—H2*A*⋯Cl1^ii^	0.96	2.82	3.688 (3)	151
C4—H4*A*⋯Cl1^ii^	0.96	2.81	3.682 (3)	152

Symmetry codes: (i) 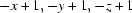; (ii) 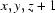.

**Fig. 1.**
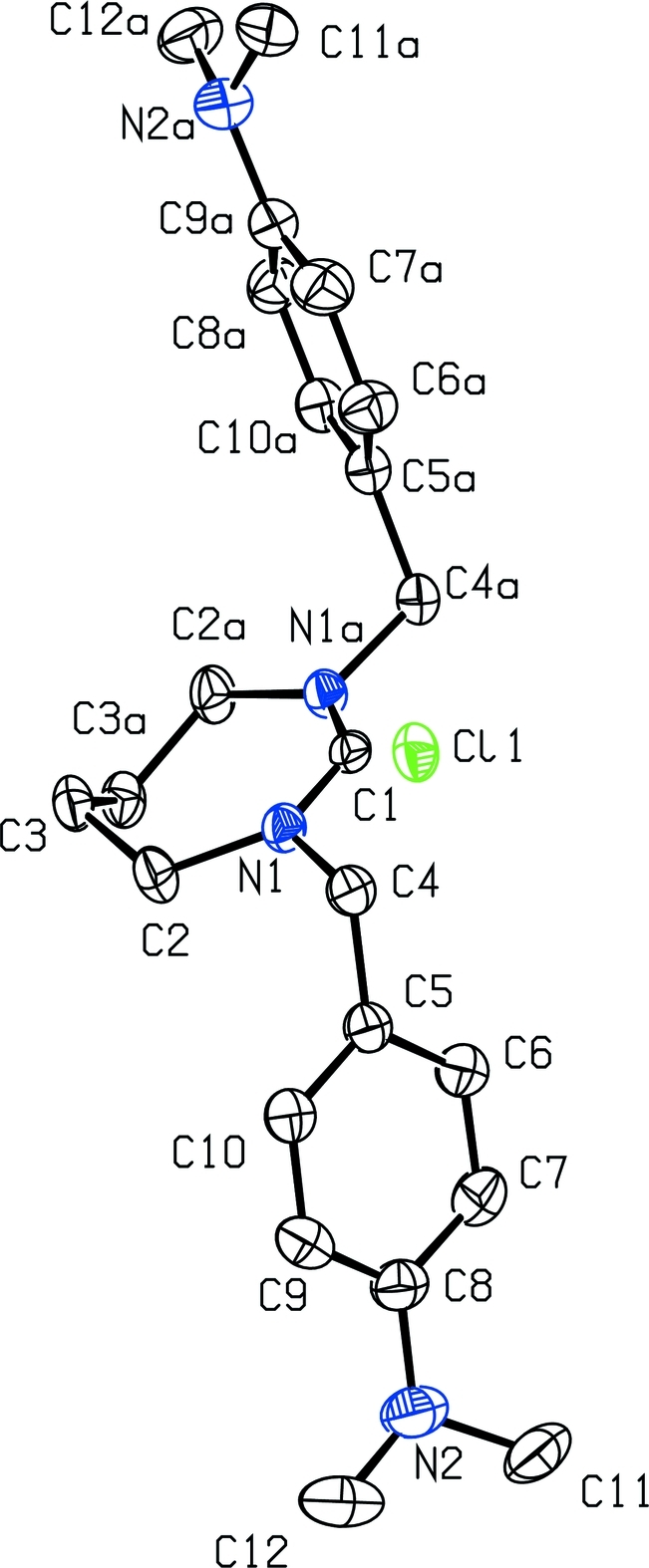
The molecular structure of the title compound, showing the atom-numbering scheme and displacement ellipsoids drawn at the 50% probability level.

**Fig. 2.**
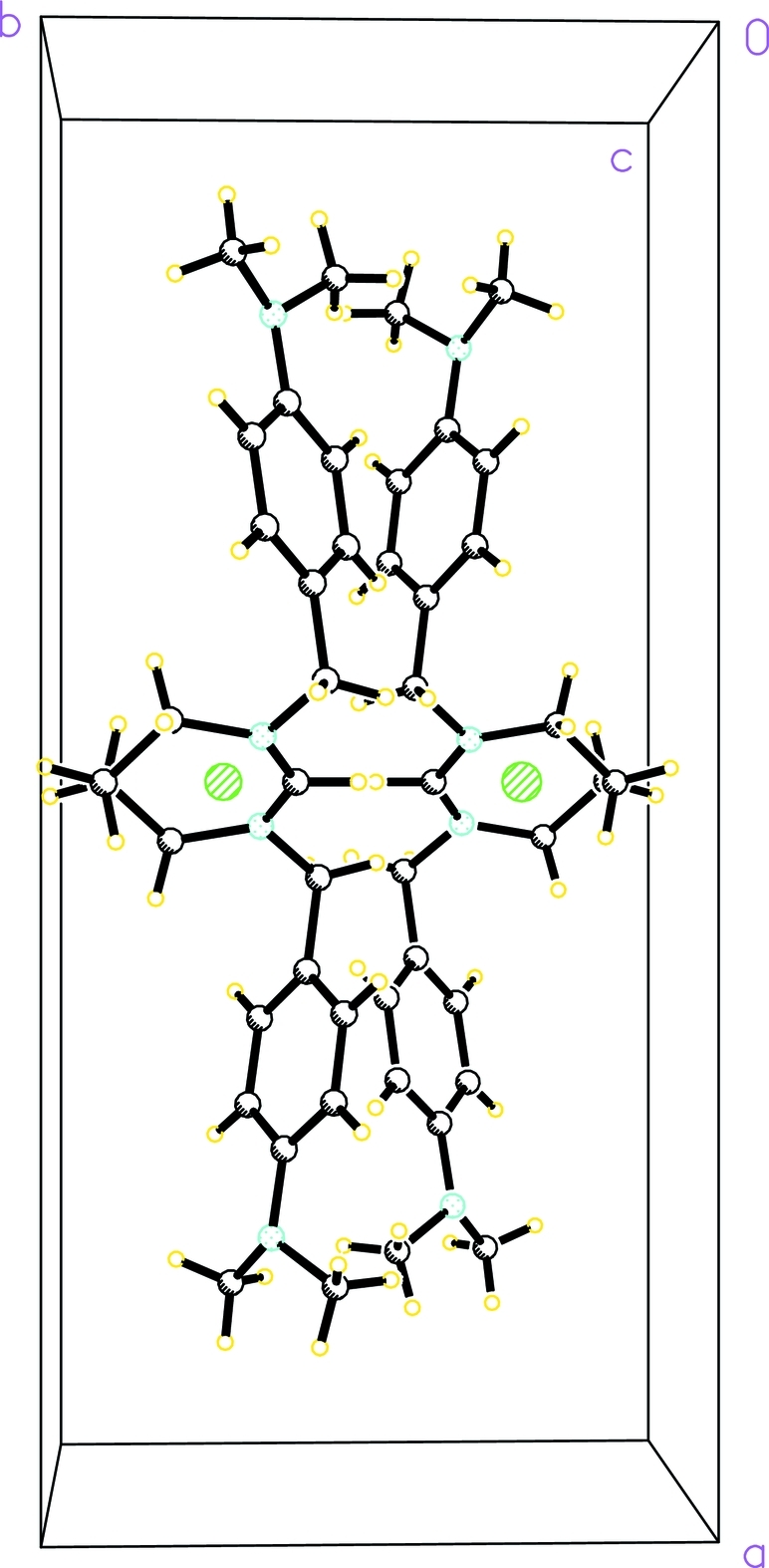
A packing diagram for (I).

**Fig. 3.**
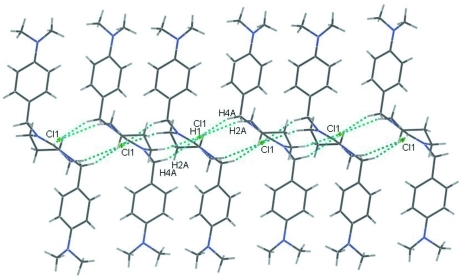
Part of the crystal structure of (I), showing the formation of linear chains of hydrogen-bonded (dashed lines) cations and anions along the [010] direction.

**Fig. 4.**
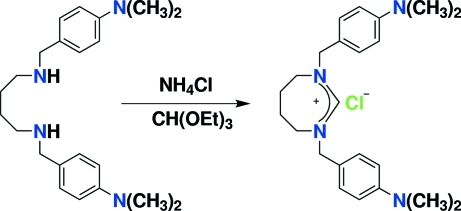
The formation of the title compound.

## supplementary crystallographic information

### Comment

*N*-heterocyclic carbenes, which can be considered phosphine mimics, have attracted considerable attentionas possible alternatives for widely used phosphine ligands (Regitz, 1996; Hermann & Köcher, 1997; Arduengo & Krafczyk, 1998; Dullius *et al.*, 1998; Evans & Boeyens, 1989; Hermann, 2002; Littke & Fu, 2002). *N*-heterocyclic carbene-containing metal complexes have also revealed excellent catalytic properties in a wide range of metal-catalyzed transformations (Glorius, 2007; Nolan, 2006). Catalysts containing these ligands are useful in Heck, Suzuki and Sonogashira couplings, Buchwald Hartwig amination, olefin metathesis, hydroformylation and hydrogenation.

In recent years, we have pursued investigations on the synthesis, characterization, crystal structure, and catalytic activities of new *N*-heterocyclic carbene derivatives (Yaşar *et al.*, 2008; Arslan *et al.*, 2007*a*, 2007*b*, 2007*c*). In the present work, we report the preparation and characterization of a novel *N*-heterocyclic carbene derivative, 1,3-*bis*(4-(dimethylamino)benzyl)-4,5,6,7-tetrahydro-1*H*-1,3-diazepin-2-ium chloride, (I). The ligand was purified by re-crystallization from an ethanol:diethylether mixture (1:2) and was characterized by elemental analysis and ^1^H and ^13^C-NMR spectroscopy. The analytical and spectroscopic data are consistent with the proposed structure given in Scheme 1.

The molecular structure of the title compound, (I), is depicted in Fig. 1. The structure consists of a 1,3-*bis*(4-(dimethylamino)benzyl)-4,5,6,7-tetrahydro-1*H*-1,3-diazepin-2-ium cation and a Cl^-^anion. All bond lengths are in normal ranges (Allen *et al.*, 1987). A seven-membered ring should have the chair, the boat, the twist chair or the twist boat according to Cremer & Pople (1975). The conformation of a seven-membered ring can be numerically described by four ring puckering parameters, *q*_2_, *q*_3_, *φ*_2_ and *φ*_3_. The 1,3-diazepane ring exhibits a puckered conformation, with puckering parameters Cremer & Pople (1975), *q*_2_= 0.374 (3) Å, *q*_3_ = 0.462 (3) Å, *φ*_2_= 347.1 (4) °, *φ*_3_= 115.7 (3)°, and *Q*_T_= 0.595 (3) Å. The largest deviations from the mean plane are 0.403 (3) Å for atoms C3 and C3A. *q*_2 _should be 0 for a 100% twist chair form. According to Cremer & Pople ring-puckering analysis results, the 1,3-diazepane seven-membered ring can be accurately described as an intermediate form between the 44% twist chair form and the 55% twist boat form.

The crystal packing is shown in Fig. 2. Although there are no intramolecular D—H···A contacts, intermolecular C—H···Cl hydrogen bonds link the molecules of (I) into one-dimensional chains extending along the [010] direction (Fig. 3, Table 1) (Macrae *et al.*, 2006).

### Experimental

To a solution of 1,4-*bis*(*p*-dimethylaminobenzylamino)butane (1 mmol) CH(OEt)_3_ (30 ml), NH_4_Cl (1 mmol) was added; the reaction mixture was heated for 18 h at 100 °C (Scheme 2). A white solid was precipitated. Then, the precipitate was crystallized from EtOH-Et_2_O (1:2) mixture (Özdemir *et al.*, 2005). 1,3-*bis*(4-(dimethylamino)benzyl)-4,5,6,7-tetrahydro-1*H*-1,3-diazepin-2-ium chloride: Yield: 3.11 g (92%), *M*.p. 247–248 °C. ^1^H NMR (300.13 MHz, DMSO) δ = 1.68 (quintet, *J* = 6.8 Hz, 4H, NCH_2_C*H*_2_C*H*_2_CH_2_N), 2.89 (s, 12H, *p*-(C*H*_3_)_2_NC_6_H_4_CH_2_), 3.52 (t, *J* = 6.8 Hz, 4H, NC*H*_2_CH_2_CH_2_C*H*_2_N), 4.54 (s, 4H, *p*-(CH_3_)_2_NC_6_H_4_C*H*_2_), 6.73 and 7.27 (d, J =8.4 Hz, 8H, *p*-(CH_3_)_2_NC_6_*H*_4_CH_2_), 8.87 (s,1*H*, 2-C*H*). ^13^C{^1^H}NMR (75.47 MHz, DMSO): δ = 24.7, 48.7 (N*C*H_2_*C*H_2_*C*H_2_*C*H_2_N), 40.7 (*p*-(*C*H_3_)_2_NC_6_H_4_CH_2_), 60.1 (*p*-(CH_3_)_2_NC_6_H_4_*C*H_2_), 112.9, 122.2, 130.1, 151.1 (*p*-(CH_3_)_2_N*C*_6_H_4_CH_2_), 158.3 (2-*C*H). Anal. Calcd. for C_23_H_33_N_4_Cl: C, 68.89; H, 8.29; N, 13.97. Found: C, 68.88; H, 8.30; N, 13.94.

### Crystal data

**Table:**

C_23_H_33_N_4_^+^·Cl^−^	*F*(000) = 864
*M**_r_* = 400.98	*D*_x_ = 1.210 Mg m^−^^3^
Orthorhombic, *P**b**c**n*	Mo *K*α radiation, λ = 0.71073 Å
Hall symbol: -P 2n 2ab	Cell parameters from 5004 reflections
*a* = 22.663 (5) Å	θ = 3.1–26.4°
*b* = 10.081 (2) Å	µ = 0.19 mm^−^^1^
*c* = 9.6368 (19) Å	*T* = 153 K
*V* = 2201.7 (8) Å^3^	Rod, colorless
*Z* = 4	0.46 × 0.12 × 0.07 mm

### Data collection

**Table:**

Rigaku Mercury CCD diffractometer	1947 independent reflections
Radiation source: Sealed Tube	1481 reflections with *I* > 2σ(*I*)
Graphite Monochromator	*R*_int_ = 0.053
Detector resolution: 14.6306 pixels mm^-1^	θ_max_ = 25.1°, θ_min_ = 3.1°
ω scans	*h* = −27→23
Absorption correction: multi-scan (REQAB; Jacobson, 1998)	*k* = −12→11
*T*_min_ = 0.918, *T*_max_ = 0.987	*l* = −11→11
14704 measured reflections	

### Refinement

**Table:**

Refinement on *F*^2^	Primary atom site location: structure-invariant direct methods
Least-squares matrix: full	Secondary atom site location: difference Fourier map
*R*[*F*^2^ > 2σ(*F*^2^)] = 0.059	Hydrogen site location: inferred from neighbouring sites
*wR*(*F*^2^) = 0.160	H-atom parameters constrained
*S* = 1.10	*w* = 1/[σ^2^(*F*_o_^2^) + (0.0629*P*)^2^ + 3.1587*P*] where *P* = (*F*_o_^2^ + 2*F*_c_^2^)/3
1947 reflections	(Δ/σ)_max_ < 0.001
130 parameters	Δρ_max_ = 0.28 e Å^−^^3^
0 restraints	Δρ_min_ = −0.30 e Å^−^^3^

### Special details

**Table:**

Geometry. All e.s.d.'s (except the e.s.d. in the dihedral angle between two l.s. planes) are estimated using the full covariance matrix. The cell e.s.d.'s are taken into account individually in the estimation of e.s.d.'s in distances, angles and torsion angles; correlations between e.s.d.'s in cell parameters are only used when they are defined by crystal symmetry. An approximate (isotropic) treatment of cell e.s.d.'s is used for estimating e.s.d.'s involving l.s. planes.
Refinement. Refinement of *F*^2^ against ALL reflections. The weighted *R*-factor *wR* and goodness of fit *S* are based on *F*^2^, conventional *R*-factors *R* are based on *F*, with *F* set to zero for negative *F*^2^. The threshold expression of *F*^2^ > σ(*F*^2^) is used only for calculating *R*-factors(gt) *etc*. and is not relevant to the choice of reflections for refinement. *R*-factors based on *F*^2^ are statistically about twice as large as those based on *F*, and *R*- factors based on ALL data will be even larger.

### Fractional atomic coordinates and isotropic or equivalent isotropic displacement parameters (Å^2^)

**Table:**

	*x*	*y*	*z*	*U*_iso_*/*U*_eq_	
Cl1	0.5000	0.27328 (9)	0.2500	0.0326 (3)	
N1	0.53058 (10)	0.3281 (2)	0.8530 (2)	0.0263 (5)	
N2	0.81399 (11)	0.3446 (3)	0.8340 (3)	0.0465 (8)	
C1	0.5000	0.3809 (4)	0.7500	0.0234 (8)	
H1	0.5000	0.4761	0.7500	0.028*	
C2	0.54103 (13)	0.1885 (3)	0.8905 (3)	0.0320 (7)	
H2A	0.5391	0.1810	0.9897	0.038*	
H2B	0.5805	0.1656	0.8631	0.038*	
C3	0.49952 (13)	0.0887 (3)	0.8287 (3)	0.0299 (7)	
H3A	0.4602	0.1074	0.8599	0.036*	
H3B	0.5100	0.0019	0.8616	0.036*	
C4	0.56701 (12)	0.4207 (3)	0.9380 (3)	0.0286 (6)	
H4A	0.5588	0.4062	1.0345	0.034*	
H4B	0.5564	0.5104	0.9160	0.034*	
C5	0.63188 (12)	0.4015 (3)	0.9123 (3)	0.0280 (6)	
C6	0.65910 (13)	0.4560 (3)	0.7968 (3)	0.0361 (7)	
H6	0.6359	0.5068	0.7327	0.043*	
C7	0.71893 (14)	0.4394 (3)	0.7709 (4)	0.0410 (8)	
H7	0.7364	0.4791	0.6902	0.049*	
C8	0.75396 (13)	0.3648 (3)	0.8622 (3)	0.0359 (7)	
C9	0.72698 (13)	0.3100 (3)	0.9785 (3)	0.0359 (7)	
H9	0.7500	0.2585	1.0425	0.043*	
C10	0.66696 (13)	0.3289 (3)	1.0031 (3)	0.0316 (7)	
H10	0.6494	0.2909	1.0846	0.038*	
C11	0.84333 (15)	0.4411 (4)	0.7441 (5)	0.0577 (11)	
H11A	0.8243	0.4424	0.6552	0.087*	
H11B	0.8840	0.4166	0.7328	0.087*	
H11C	0.8410	0.5276	0.7854	0.087*	
C12	0.85025 (15)	0.2829 (4)	0.9408 (4)	0.0569 (11)	
H12A	0.8501	0.3374	1.0225	0.085*	
H12B	0.8900	0.2737	0.9074	0.085*	
H12C	0.8346	0.1970	0.9628	0.085*	

### Atomic displacement parameters (Å^2^)

**Table:**

	*U*^11^	*U*^22^	*U*^33^	*U*^12^	*U*^13^	*U*^23^
Cl1	0.0410 (6)	0.0223 (5)	0.0347 (5)	0.000	0.0031 (4)	0.000
N1	0.0280 (12)	0.0207 (11)	0.0302 (12)	−0.0008 (9)	0.0021 (10)	−0.0010 (10)
N2	0.0300 (14)	0.0458 (17)	0.064 (2)	−0.0016 (12)	0.0035 (13)	−0.0117 (15)
C1	0.0210 (17)	0.0196 (18)	0.030 (2)	0.000	0.0053 (16)	0.000
C2	0.0399 (16)	0.0206 (14)	0.0356 (16)	0.0035 (12)	−0.0043 (13)	0.0019 (12)
C3	0.0376 (15)	0.0191 (13)	0.0331 (16)	0.0013 (12)	0.0030 (13)	0.0035 (11)
C4	0.0303 (14)	0.0239 (14)	0.0315 (15)	0.0006 (11)	0.0013 (12)	−0.0053 (12)
C5	0.0300 (15)	0.0251 (14)	0.0290 (15)	−0.0003 (11)	0.0000 (11)	−0.0032 (12)
C6	0.0331 (16)	0.0372 (17)	0.0379 (17)	0.0004 (13)	0.0013 (13)	0.0056 (14)
C7	0.0386 (17)	0.0415 (18)	0.0430 (19)	−0.0055 (14)	0.0073 (14)	0.0058 (15)
C8	0.0299 (15)	0.0327 (16)	0.0450 (17)	−0.0011 (12)	0.0007 (14)	−0.0093 (14)
C9	0.0353 (16)	0.0350 (17)	0.0373 (17)	0.0069 (13)	−0.0062 (13)	−0.0036 (14)
C10	0.0348 (16)	0.0299 (15)	0.0300 (15)	−0.0002 (12)	0.0000 (12)	−0.0013 (13)
C11	0.0358 (18)	0.045 (2)	0.092 (3)	−0.0136 (15)	0.0185 (19)	−0.014 (2)
C12	0.0326 (17)	0.067 (3)	0.071 (3)	0.0097 (17)	−0.0076 (18)	−0.024 (2)

### Geometric parameters (Å, °)

**Table:**

N1—C1	1.322 (3)	C5—C6	1.386 (4)
N1—C2	1.472 (3)	C5—C10	1.391 (4)
N1—C4	1.491 (3)	C6—C7	1.389 (4)
N2—C8	1.402 (4)	C6—H6	0.9600
N2—C12	1.456 (5)	C7—C8	1.404 (5)
N2—C11	1.462 (5)	C7—H7	0.9600
C1—N1^i^	1.322 (3)	C8—C9	1.391 (4)
C1—H1	0.9600	C9—C10	1.394 (4)
C2—C3	1.501 (4)	C9—H9	0.9600
C2—H2A	0.9600	C10—H10	0.9600
C2—H2B	0.9600	C11—H11A	0.9599
C3—C3^i^	1.517 (6)	C11—H11B	0.9599
C3—H3A	0.9600	C11—H11C	0.9599
C3—H3B	0.9600	C12—H12A	0.9599
C4—C5	1.503 (4)	C12—H12B	0.9599
C4—H4A	0.9600	C12—H12C	0.9599
C4—H4B	0.9600		
			
C1—N1—C2	130.8 (3)	C10—C5—C4	121.5 (3)
C1—N1—C4	116.8 (2)	C5—C6—C7	122.1 (3)
C2—N1—C4	112.0 (2)	C5—C6—H6	119.0
C8—N2—C12	118.2 (3)	C7—C6—H6	119.0
C8—N2—C11	117.3 (3)	C6—C7—C8	120.3 (3)
C12—N2—C11	116.5 (3)	C6—C7—H7	119.9
N1^i^—C1—N1	132.6 (4)	C8—C7—H7	119.9
N1^i^—C1—H1	113.7	C9—C8—N2	121.7 (3)
N1—C1—H1	113.7	C9—C8—C7	118.0 (3)
N1—C2—C3	116.3 (2)	N2—C8—C7	120.3 (3)
N1—C2—H2A	108.2	C8—C9—C10	120.8 (3)
C3—C2—H2A	108.2	C8—C9—H9	119.6
N1—C2—H2B	108.2	C10—C9—H9	119.6
C3—C2—H2B	108.2	C5—C10—C9	121.5 (3)
H2A—C2—H2B	107.4	C5—C10—H10	119.2
C2—C3—C3^i^	112.8 (2)	C9—C10—H10	119.2
C2—C3—H3A	109.0	N2—C11—H11A	109.5
C3^i^—C3—H3A	109.0	N2—C11—H11B	109.5
C2—C3—H3B	109.0	H11A—C11—H11B	109.5
C3^i^—C3—H3B	109.0	N2—C11—H11C	109.5
H3A—C3—H3B	107.8	H11A—C11—H11C	109.5
N1—C4—C5	111.8 (2)	H11B—C11—H11C	109.5
N1—C4—H4A	109.3	N2—C12—H12A	109.5
C5—C4—H4A	109.3	N2—C12—H12B	109.5
N1—C4—H4B	109.3	H12A—C12—H12B	109.5
C5—C4—H4B	109.3	N2—C12—H12C	109.5
H4A—C4—H4B	107.9	H12A—C12—H12C	109.5
C6—C5—C10	117.4 (3)	H12B—C12—H12C	109.5
C6—C5—C4	121.1 (3)		
			
C2—N1—C1—N1^i^	−0.7 (2)	C12—N2—C8—C9	−10.0 (4)
C4—N1—C1—N1^i^	171.0 (2)	C11—N2—C8—C9	−157.9 (3)
C1—N1—C2—C3	−18.0 (4)	C12—N2—C8—C7	171.5 (3)
C4—N1—C2—C3	170.0 (2)	C11—N2—C8—C7	23.6 (5)
N1—C2—C3—C3^i^	59.9 (4)	C6—C7—C8—C9	−0.5 (5)
C1—N1—C4—C5	−109.3 (2)	C6—C7—C8—N2	178.0 (3)
C2—N1—C4—C5	63.9 (3)	N2—C8—C9—C10	−178.6 (3)
N1—C4—C5—C6	80.0 (3)	C7—C8—C9—C10	−0.1 (5)
N1—C4—C5—C10	−100.1 (3)	C6—C5—C10—C9	−0.8 (4)
C10—C5—C6—C7	0.2 (5)	C4—C5—C10—C9	179.3 (3)
C4—C5—C6—C7	−179.9 (3)	C8—C9—C10—C5	0.7 (5)
C5—C6—C7—C8	0.4 (5)		

Symmetry codes: (i) −*x*+1, *y*, −*z*+3/2.

### Hydrogen-bond geometry (Å, °)

**Table:**

*D*—H···*A*	*D*—H	H···*A*	*D*···*A*	*D*—H···*A*
C1—H1···Cl1^ii^	0.96	2.53	3.486 (4)	180
C2—H2A···Cl1^iii^	0.96	2.82	3.688 (3)	151
C4—H4A···Cl1^iii^	0.96	2.81	3.682 (3)	152

Symmetry codes: (ii) −*x*+1, −*y*+1, −*z*+1; (iii) *x*, *y*, *z*+1.

## References

[bb1] Allen, F. H., Kennard, O., Watson, D. G., Brammer, L., Orpen, A. G. & &Taylor, R. (1987). *J. Chem. Soc. Perkin Trans. 2*, pp. S1–19.

[bb2] Arduengo, A. J. & Krafczyk, R. (1998). *Chem. Unserer. Z.* **32**, 6–14.

[bb3] Arslan, H., VanDerveer, D., Özdemir, İ., Demir, S. & Çetinkaya, B. (2007*a*). *Acta Cryst.* E**63**, m770–m771.

[bb4] Arslan, H., VanDerveer, D., Yaşar, S., Özdemir, I. & Çetinkaya, B. (2007*b*). *Acta Cryst.* E**63**, m942–m944.

[bb5] Arslan, H., VanDerveer, D., Yaşar, S., Özdemir, İ. & Çetinkaya, B. (2007*c*). *Acta Cryst.* E**63**, m1001–m1003.

[bb6] Cremer, D. & Pople, J. A. (1975). *J. Am. Chem. Soc.* **97**, 1354–1358.

[bb7] Dullius, J. E. L., Suarez, P. A. Z., Einloft, S., de Souza, R. F., Dupont, J., Fischer, J. & De Cian, A. (1998). *Organometallics*, **17**, 815-819.

[bb8] Evans, D. G. & Boeyens, J. C. A. (1989). *Acta Cryst.* B**45**, 581–590.

[bb9] Glorius, F. (2007). *Topics in Organometallic Chemistry*, Vol. 21, *N-Heterocyclic Carbenes inTransition Metal Catalysis* Heidelberg: Springer.

[bb10] Hermann, W. A. (2002). *Angew. Chem. Int. Ed.* **41**, 1290–1309.

[bb11] Hermann, W. A. & Köcher, C. (1997). *Angew. Chem. Int. Ed. Engl.* **36**, 2162–2187.

[bb12] Jacobson, R. (1998). *REQAB.* Molecular Structure Corporation, The Woodlands, Texas,USA.

[bb13] Littke, A. F. & Fu, G. C. (2002). *Angew. Chem. Int. Ed.* **41**, 4176–4211. 10.1002/1521-3773(20021115)41:22<4176::AID-ANIE4176>3.0.CO;2-U 12434342

[bb14] Macrae, C. F., Edgington, P. R., McCabe, P., Pidcock, E., Shields, G. P., Taylor, R., Towler, M. & van de Streek, J. (2006). *J. Appl. Cryst.* **39**, 453–457.

[bb15] Nolan, S. P. (2006). *N-HeterocyclicCarbenes in Synthesis* Weinheim: Wiley.

[bb16] Özdemir, I., Gürbüz, N., Gök, Y., Çetinkaya, E. & Çetinkaya, B. (2005). *Synlett*, **15**, 2394–2396.

[bb17] Regitz, M. (1996). *Angew. Chem. Int. Ed. Engl.* **35**, 725–728.

[bb18] Rigaku/MSC (2001). *CrystalClear* Rigaku/MSC, The Woodlands, Texas, USA.

[bb19] Sheldrick, G. M. (2008). *Acta Cryst.* A**64**, 112–122. 10.1107/S0108767307043930 18156677

[bb20] Yaşar, S., Özdemir, I., Çetinkaya, B., Renaud, J. & Bruneau, L. (2008). *Eur. J. Org. Chem.* **12**, 2142–2149.

